# ﻿Another new species of the genus *Habrophlebia* Eaton, 1881 (Ephemeroptera, Leptophlebiidae) from the Maghreb

**DOI:** 10.3897/zookeys.1186.112796

**Published:** 2023-12-11

**Authors:** Majida El Alami, Laurent Vuataz, Sara El Yaagoubi, Michel Sartori

**Affiliations:** 1 Université Abdelmalek Essaâdi, Faculté des Sciences, Département de Biologie, Laboratoire Ecologie, Systématique et Conservation de la Biodiversité (LESB), Unité de Recherche Labellisée CNRST N°18. B.P.2121. Tétouan 93002, Morocco Université Abdelmalek Essaâdi Tétouan Morocco; 2 Muséum Cantonal des Sciences Naturelles, Département de Zoologie, Palais de Rumine, Place Riponne 6, CH-1005, Lausanne, Switzerland Muséum Cantonal des Sciences Naturelles, Département de Zoologie Lausanne Switzerland; 3 University of Lausanne (UNIL), Department of Ecology and Evolution, CH-1015 Lausanne, Switzerland University of Lausanne (UNIL) Lausanne Switzerland

**Keywords:** COI, *Habrophlebiadakkii* sp. nov., mayflies, Morocco, Rif Mountains, Spain, systematics, West Palaearctic

## Abstract

A new species belonging to the genus *Habrophlebia* Eaton, 1881 is described at the nymphal stage from the Rif Mountains of Morocco. This species presents unique features, such as the chorionic arrangement of the egg and the ornamentation of the posterior margin of abdominal tergites. It is compared to all west European *Habrophlebia* species and a table with discriminating characters is given. A phylogenetic reconstruction based on COI sequences fully supports the hypothesis of a new species in the Rif Mountains, with possible further distribution in southern Spain.

## ﻿Introduction

Affiliated to the subfamily Habrophlebiinae (Leptophlebiidae), *Habrophlebia* Eaton, 1881 is a Holarctic genus, represented by a single species in the Nearctic (Peters 1979) and eight in the West Palearctic (Jacob and Sartori 1984; Alba-Tercedor 2000; Bauernfeind and Soldán 2012; Barber-James et al. 2013; Benhadji et al. 2018; Kechemir et al. 2020). So far, five species of this genus have been reported from North Africa: *Habrophlebiavaillantorum* Thomas, 1986 (Thomas et al. 1999) found in the Moroccan High Atlas; *H.consiglioi* Biancheri, 1959, described from Sardinia and recently reported from Tunisia (Zrelli et al. 2011, 2016); two species discovered in northern Algeria, *H.hassainae* Benhadji & Sartori, 2018 and *H.djurdjurensis* Kechemir, Sartori & Lounaci, 2020; the presence of *H.fusca* (Curtis, 1834) in the Maghreb (Dakki and El Agbani 1983; Giudicelli and Dakki 1984; Dakki 1987; El Agbani et al. 1992; Lounaci et al. 2000) remains doubtful (Thomas 1998; Thomas et al. 1999) and unconfirmed (El Alami 2002; Benhadji et al. 2018; Kechemir et al. 2020; El Alami et al. 2022a). In fact, in northern Morocco, a review of the ephemeropteran collection from the Rif Mountains, whose specimens come from various streams, revealed the absence of the species in this biogeographical area (El Alami et al. 2022a).

With the exception of *H.fusca*, *H.lauta* McLachlan, 1884 and *H.eldae* Jacob & Sartori, 1984, the majority of species have a very restricted distribution. *Habrophlebiaantoninoi* Alba-Tercedor, 2000, is a southern Iberian endemic, *H.vaillantorum* is a Moroccan High Atlas endemic, *H.hassainae* and *H.djurdjurensis* are Algerian endemics and *H.consiglioi* was collected only in Sardinia and Tunisia.

The isolation of populations in West Palearctic rivers and streams due to geographical barriers has favored speciation within the genus and contributed to an increase in the endemism rate within its biogeographical zone (Dakki 1987; Cheylan 1990; El Alami et al. 2022a).

The Rif is the most northerly mountain range in Morocco. It is characterized by a number of features that give its aquatic fauna a certain originality (Bennas 2002; El Alami 2002; Blondel et al. 2010; Hajji 2013; Errochdi et al. 2014; El Bazi et al. 2017; Khadri et al. 2017). Although the climate is typically Mediterranean, the western part is subject to Atlantic disturbances and is much wetter than the eastern part. In addition, the geological history of the Rif presents two distinctive features: the dominance of schistose or marly soils, with a limestone formation that extends mainly to the west (Thauvin 1971a, b; El Gharbaoui 1981), and the generally steep relief resulting in valleys that are often deep. The connections between the Rif and the Iberian Peninsula over their geological history have enabled a fauna exchange between the African and European continents and enriched the region’s populations (Bonada et al. 2009; Jaskuła 2015; Poulakakis et al. 2015; Mabrouki et al. 2017; Múrria et al. 2017; Bennas et al. 2018; Slimani 2018; Taybi et al. 2020; El Alami et al. 2022a).

Ongoing research on mayflies from northern Morocco has revealed that the *Habrophlebia* specimens are not related to either *H.vaillantorum* or *H.fusca*. They belong in fact to a new species that has been cited as *Habrophlebia* sp. in numerous works on the Rif (El Alami 2002; El Bazi et al. 2017; Khadri et al. 2017; Guellaf et al. 2021; El Alami et al. 2022a).

The main objective of the present study is to describe this species based on Mrs El Alami’s collection and from material freshly collected by Ms El Yaagoubi. Morphological and molecular data (COI sequences) were combined to separate the nymphs of the Rif populations from other western Palearctic species. We also provide preliminary information on its distribution and ecological preferences.

## ﻿Materials and methods

**Figure 1.**
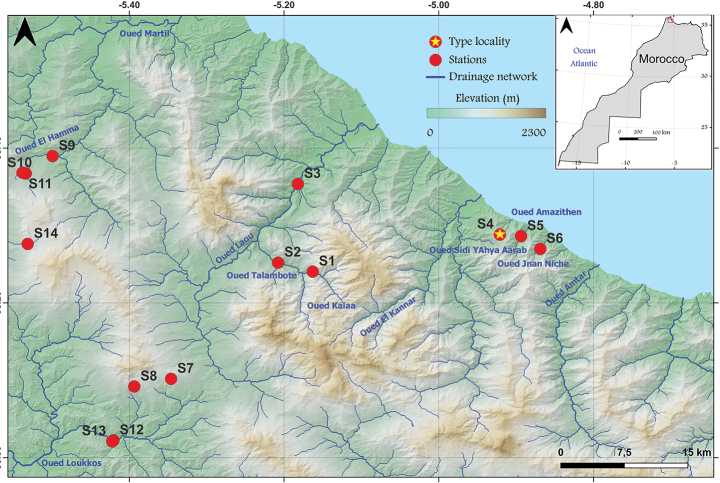
The sampling site localization of *Habrophlebiadakkii* sp. nov. in the Rif domain.

Samplings were performed by the LESCB team between 1997 and 2023 (Fig. 1). They were subsequently preserved in 70% or 95% ethanol for description and DNA extraction.

Pictures of nymphal habitus were made using a Canon EOS 6D camera and the Visionary Digital Passport imaging system (formerly available and distributed by Dun Inc., Virginia), and processed with Adobe Photoshop Lightroom and Helicon Focus ver. 5.3.

Nymphal dissection was performed in Cellosolve or in 10% KOH, and specimens were mounted on slides with Euparal medium, or the dissected parts of the nymphs were mounted directly in Hoyer’s liquid (Alba-Tercedor 1988).

Microscopic pictures were taken using an Olympus BX51 microscope coupled with an Olympus SC50 camera; pictures were enhanced with the stacking software Olympus Stream Basic ver. 2.3.2. and Adobe Photoshop ver. 21.2.2. Alternatively, pictures were taken using an Olympus CX41 microscope.

### ﻿Molecular analysis

To complement our morphological investigations, we sequenced a 658 bp fragment of the mitochondrial gene cytochrome oxidase subunit 1 (COI hereafter) for specimens of the new species and other *Habrophlebia* species collected in the Maghreb. For this, the DNA extraction method described in Vuataz et al. (2011) was used to ensure non-destructive extraction. The Polymerase Chain Reaction (PCR), purification and sequencing steps followed the method outlined in El Alami et al. (2022b). Forward and reverse sequencing reads were assembled and edited in CodonCode Aligner ver. 10.0.2 (Codon-Code Corporation, Dedham, MA). To augment our molecular dataset, we initially downloaded all COI sequences associated with *Habrophlebia* available on the GenBank database as of 27 August 2023, totaling 69 records. Additionally, we obtained *Habrophlebia* sequences from the BOLDSYSTEMS data portal on the same date and selectively retained only those not shared with GenBank, yielding an additional set of six sequences. We then manually excluded GenBank/BOLD sequences obtained from specimens collected outside the western Palearctic region or shorter than 250 bp. This selection process was conducted after confirming that the COI sequences of the new species were clearly distinct from the removed sequences (data not shown). We also included sequences from the Freshwater Diversity Identification for Europe (FREDIE) project (unpublished; https://wp.fredie.eu/). A total of 63 sequences remained for further analyses, comprising 18 newly generated sequences (Table 1), 38 sequences from GenBank [five from Cardoni et al. (2015); ten from Gattolliat et al. (2015); 15 from Morinière et al. (2017); three from Behrens-Chapuis et al. (2021); five unpublished International Barcode of Life (iBOL) data releases], five from BOLD (BGMAY026-10, BGMAY092-11, BGMAY446-11, DTNHM444-21, TRSKA4318-20), and two from FREDIE: MO008a_SR4H12 (Morocco, Rif, wadi Farda at Imizzar) and ES035a_SR4E11 (Spain, Cordilleras Béticas, Barranco de los Madroñales near Otivar). Two sequences from specimens of *Habroleptoides* Schönemund, 1929 were downloaded from GenBank and included in the dataset as outgroups. All sequences were aligned using MAFFT (Katoh et al. 2019) with default settings as implemented in Jalview ver. 2.11.2.7 (Waterhouse et al. 2009). The number of parsimony-informative sites of the alignment was calculated in Mega ver. 10.2.4 (Kumar et al. 2018; Stecher et al. 2020).

**Table 1.** Newly sequenced specimens (nymphs) for the present study, with collection information, GenBank accession numbers and nomenclature details.

Specimen catalogue nb	Species	Country	Stage	Locality	GPS coordinates	Date	GenBank ID	GenSeq Nomenclature
GBIFCH01144259	*Habrophlebiadakkii* sp. nov.	Morocco	Nymph	Sidi Yahia Aarab	35°17.179'N, 4°53.625'W	27.xi.2021	OR570530	genseq-2 COI
GBIFCH01144258	*Habrophlebiadakkii* sp. nov.	Morocco	Nymph	El Ouesteyine	35°17.299'N, 4°55.267'W	1.ix.2021	OR570531	genseq-2 COI
GBIFCH01144262	*Habrophlebiadakkii* sp. nov.	Morocco	Nymph	Beni idder	35°22.102'N, 5°32.283'W	16.vii.2021	OR570532	genseq-2 COI
GBIFCH00970948	*Habrophlebiadakkii* sp. nov.	Morocco	Nymph	Tanaqoub	35°5.533'N, 5°23.604'W	31.iii.2021	OR570533	genseq-2 COI
GBIFCH01144257	*Habrophlebiadakkii* sp. nov.	Morocco	Nymph	Mezine village	35°6.133'N, 5°20.767'W	31.iii.2021	OR570534	genseq-2 COI
GBIFCH00970944	*Habrophlebiadakkii* sp. nov.	Morocco	Nymph	Jbel Laalam	35°23.387'N, 5°29.953'W	20.iv.2021	OR570535	genseq-2 COI
GBIFCH00970947	*Habrophlebiadakkii* sp. nov.	Morocco	Nymph	Souk El Had	35°1.283'N, 5°25.300'W	11.iv.2021	OR570536	genseq-2 COI
GBIFCH00970945	*Habrophlebiadakkii* sp. nov.	Morocco	Nymph	Tzroute	35°16.583'N, 5°31.883'W	2.v.2021	OR570537	genseq-2 COI
GBIFCH01144261	*Habrophlebiadakkii* sp. nov.	Morocco	Nymph	Tzroute	35°16.583'N, 5°31.883'W	2.v.2021	OR570538	genseq-2 COI
GBIFCH00970946	*Habrophlebiadakkii* sp. nov.	Morocco	Nymph	Hammadesh	35°22.033'N, 5°32.033'W	20.iv.2021	OR570539	genseq-2 COI
GBIFCH00970949	*Habrophlebia* sp. 2	Morocco	Nymph	Afeska	35°10.184'N, 5°13.105'W	2.iv.2021	OR570540	genseq-4 COI
79JJ30_B07	*Habrophlebiahassainae*	Algeria	Nymph	El Ourit	34°51'57’’N, 1°15'54’’W	1.i.2016	OR570541	genseq-4 COI
79JJ30_G06	*Habrophlebiahassainae*	Algeria	Nymph	El Ourit	34°51'57"N, 1°15'54"W	5.ii.2016	OR570542	genseq-4 COI
GBIFCH00673196	*Habrophlebiadjurdjurensis*	Algeria	Nymph	Tirourda	36°29.431'N, 4°21.693'E	9.vii.2019	OR570543	genseq-4 COI
GBIFCH00673194	*Habrophlebiadjurdjurensis*	Algeria	Nymph	Echemlili	36°28.267'N, 3°59.84'E	25.v.2018	OR570544	genseq-4 COI
GBIFCH00673195	*Habrophlebiadjurdjurensis*	Algeria	Nymph	Echemlili	36°28.267'N, 3°59.84'E	25.v.2018	OR570545	genseq-4 COI
GBIFCH00673199	*Habrophlebiadjurdjurensis*	Algeria	Nymph	Ouadhias	36°29.279'N, 4°07.362'E	9.vii.2019	OR570546	genseq-4 COI
GBIFCH01211557	*Habrophlebiadjurdjurensis*	Algeria	Nymph	Tala Rana Selloum 2	36°26.902'N, 4°18.820'E	28.iv.2021	OR570547	genseq-4 COI

To explore and visualize the COI evolutionary divergence, we employed pairwise genetic distances and gene tree approaches. COI pairwise distances were calculated using the dist.dna function from the ape 5.7-1 package (Paradis and Schliep 2019) in R ver. 4.3.1 (R Core Team 2023), selecting the raw model and the pairwise.deletion option, corresponding to uncorrected p-distances (see Srivathsan and Meier 2012) with missing data removed in a pairwise way. Mean, minimum, and maximum distances within and between putative COI species, referred to as Molecular Operational Taxonomic Units (MOTUs) hereafter, were calculated using the ddply function from the plyr ver. 1.8.8 package (Wickham 2011). The assignment of COI sequences to MOTUs was determined based on the results of the species delimitation analyses (as described below). Prior to reconstructing the COI gene tree, the best evolutionary model (GTR+Γ) was selected based on the second-order Akaike information criterion (AICc; Hurvich and Tsai 1989) implemented in JmodelTest ver. 2.1.10 (Darriba et al. 2012) with five substitution schemes, six gamma categories and default values for other parameters. To account for different substitution rates among COI codon positions, we analyzed our data set in two partitions, one with first and second codon positions, and the other with third positions (1 + 2, 3). Bayesian inference analysis was performed using BEAST ver. 1.10.4 (Suchard et al. 2018) on the CIPRES Science Gateway ver. 3.3 (Miller et al. 2010). The input BEAST file was generated in BEAUTi ver. 1.10.4 (Suchard et al. 2018), incorporating the selected evolutionary model and partition scheme described above. A relaxed molecular clock model (uncorrelated lognormal) and a UPGMA starting tree were used, with default settings for other parameters. Two independent Markov chain Monte Carlo (MCMC) analyses were run for 50 million generations, logging parameters every 1000 generations. Convergence of the MCMC runs was visually verified in Tracer ver. 1.7.2 (Rambaut et al. 2018). The log and tree files from the independent runs were combined using LogCombiner ver. 1.10.4 (Suchard et al. 2018), after discarding the initial 10% of trees as burn-in, ensuring that all parameters reached effective sample size values > 200. The maximum clade credibility tree was obtained using TreeAnnotator ver. 1.10.4 (Suchard et al. 2018) with default settings and then visualized and edited in iTOL ver. 6.8 (Letunic and Bork 2021).

Finally, we applied three contrasting single-locus species delimitation methods to our COI dataset: the distance-based ASAP (Assemble Species by Automatic Partitioning; Puillandre et al. 2020), and the tree-based GMYC (General Mixed Yule-Coalescent; Pons et al. 2006; Fujisawa and Barraclough 2013) and mPTP (multi-rate Poisson Tree Processes; Kapli et al. 2017) approaches. ASAP, an improved version of the ABGD (Automatic Barcode Gap Discovery; Puillandre et al. 2012) approach, was employed using the ASAP webserver (https://bioinfo.mnhn.fr/abi/public/asap/asapweb.html), computing the genetic distances from our COI alignment under simple p-distances with all other settings set to default. The GMYC model, which requires a time-calibrated ultrametric tree as input, implements a maximum likelihood (ML) approach that defines a threshold separating the branches modelled under speciation events (Yule process) from those described by allele neutral coalescence. The ultrametric tree used as input for GMYC was generated in BEAST, following the same procedure described earlier. However, a reduced dataset was utilized, in which outgroups were excluded and haplotypes were pruned (see Talavera et al. 2013) using Collapsetypes ver. 4.6 (Chesters 2013). MCMC chains were run here for a total of 30 million generations. GMYC was run in R using the SPLITS package 1.0–20 (Ezard et al. 2009). We favored the single-threshold version of the GMYC model because it was shown to outperform the multiple-threshold version (Fujisawa and Barraclough 2013). The mPTP approach, an extension of the PTP method by Zhang et al. (2013), also exploits phylogenetic differences within and between species, but with the advantage of directly using the number of substitutions from a phylogenetic tree, eliminating the need for time calibration. The input ML tree for mPTP was generated in RAxML-NG ver. 1.1.0 (Kozlov et al. 2019), selecting the all-in-one (ML search + bootstrapping) option and MRE-based bootstrap convergence criterion. The best model of evolution and the partition scheme specified above, as well as 50 random and 50 parsimony starting trees, were implemented.

### ﻿Abbreviations

**MZL**Muséum Cantonal des Sciences Naturelles, Lausanne (Switzerland);

**LESCB** Laboratoire d’Ecologie, Systématique et Conservation de la Biodiversité (Morocco).

## ﻿Results

### ﻿Systematics

#### ﻿ Habrophlebia dakkii

Taxon classificationAnimaliaEphemeropteraLeptophlebiidae

El Alami, Sartori & Vuataz sp. nov.

0DC4D940-8F29-5037-9C57-5C3C5357C9FC

https://zoobank.org/D4B71565-041B-417A-8575-8348702DCF73

**Figure 2.**
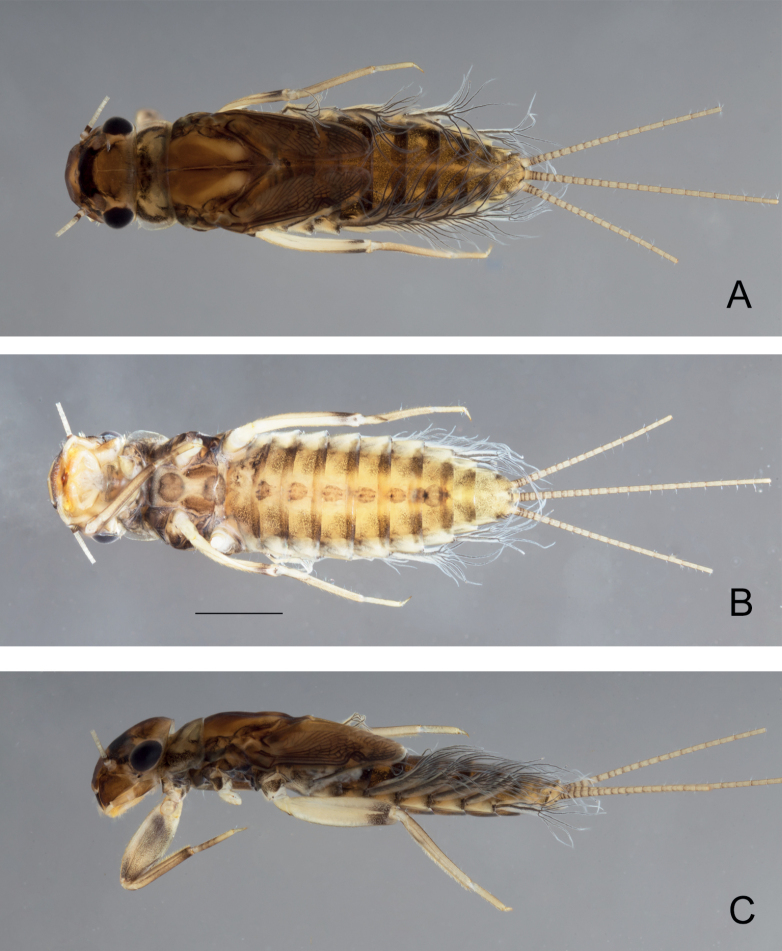
*Habrophlebiadakkii* sp. nov., habitus of the nymph **A** dorsal view **B** ventral view **C** lateral view. Scale bar: 1 mm.

**Figure 3.**
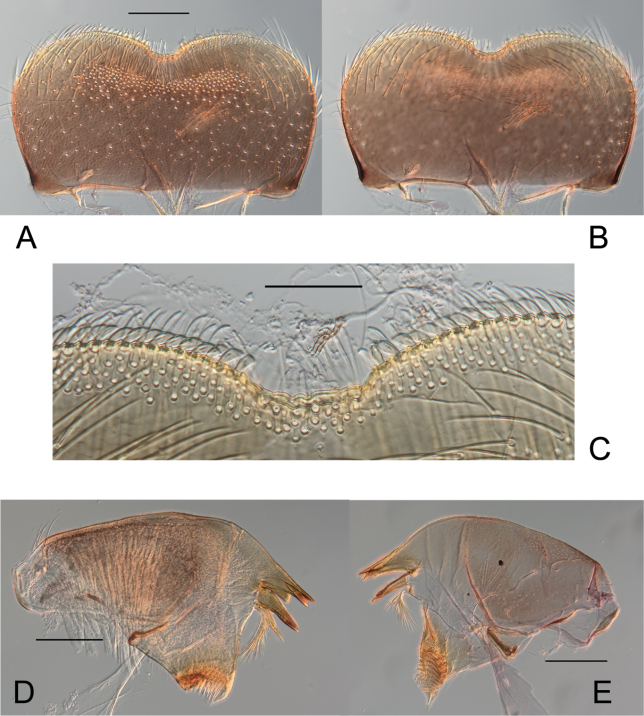
*Habrophlebiadakkii* sp. nov., nymphal mouthparts **A** labrum, dorsal view **B** labrum, ventral view **C** emargination of the labrum **D** left mandible **E** right mandible. Scale bars: 100 µm (**A, B**), 50 µm (**C**), 200 µm (**D, E**).

**Figure 4.**
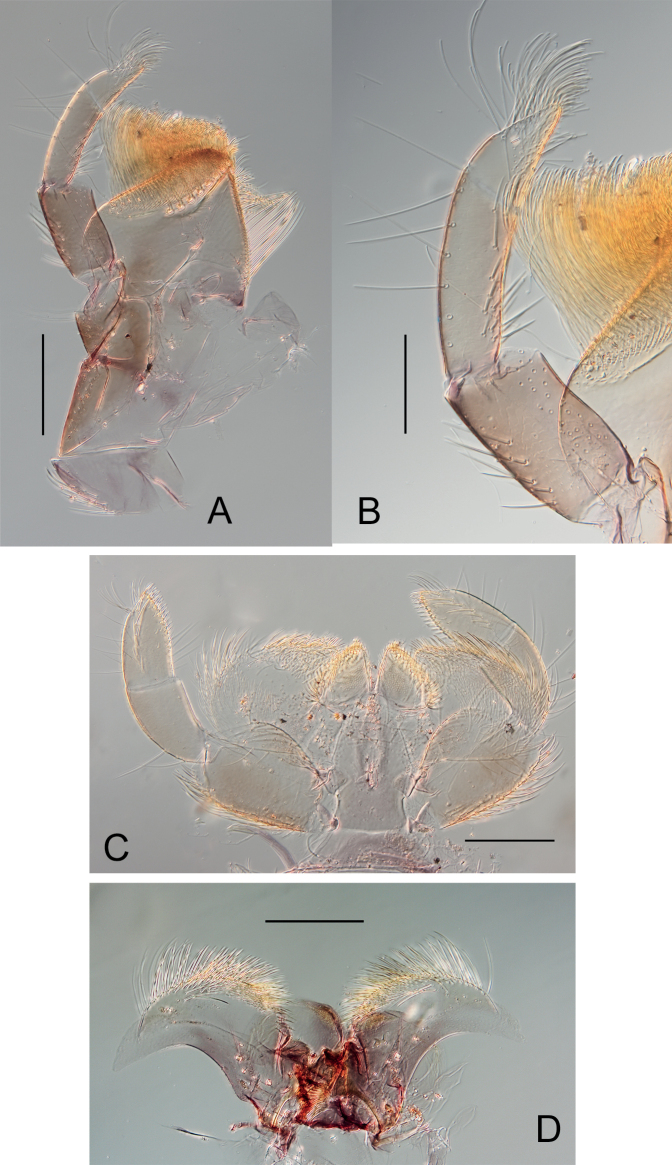
*Habrophlebiadakkii* sp. nov., nymphal mouthparts **A** maxilla **B** maxillary palp **C** labium **D** hypopharynx. Scale bars: 200 µm (**A, C, D**), 100 µm (**B**).

**Figure 5.**
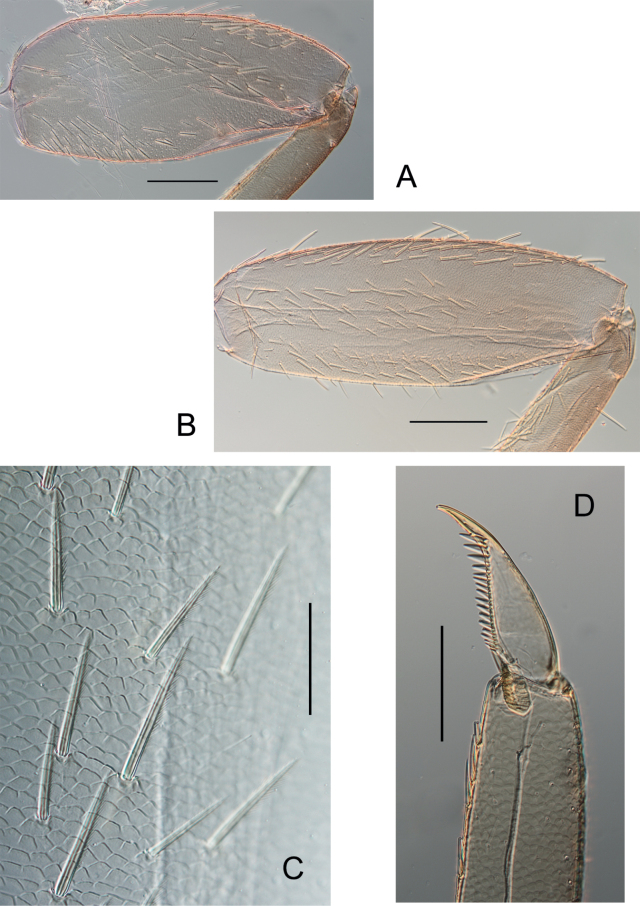
*Habrophlebiadakkii* sp. nov., nymphal legs **A** fore femur **B** hind femur **C** setae on upper face of hind femur **D** claw. Scale bars: 200 µm (**A, B**); 50 µm (**C**); 100 µm (**D**).

**Figure 6.**
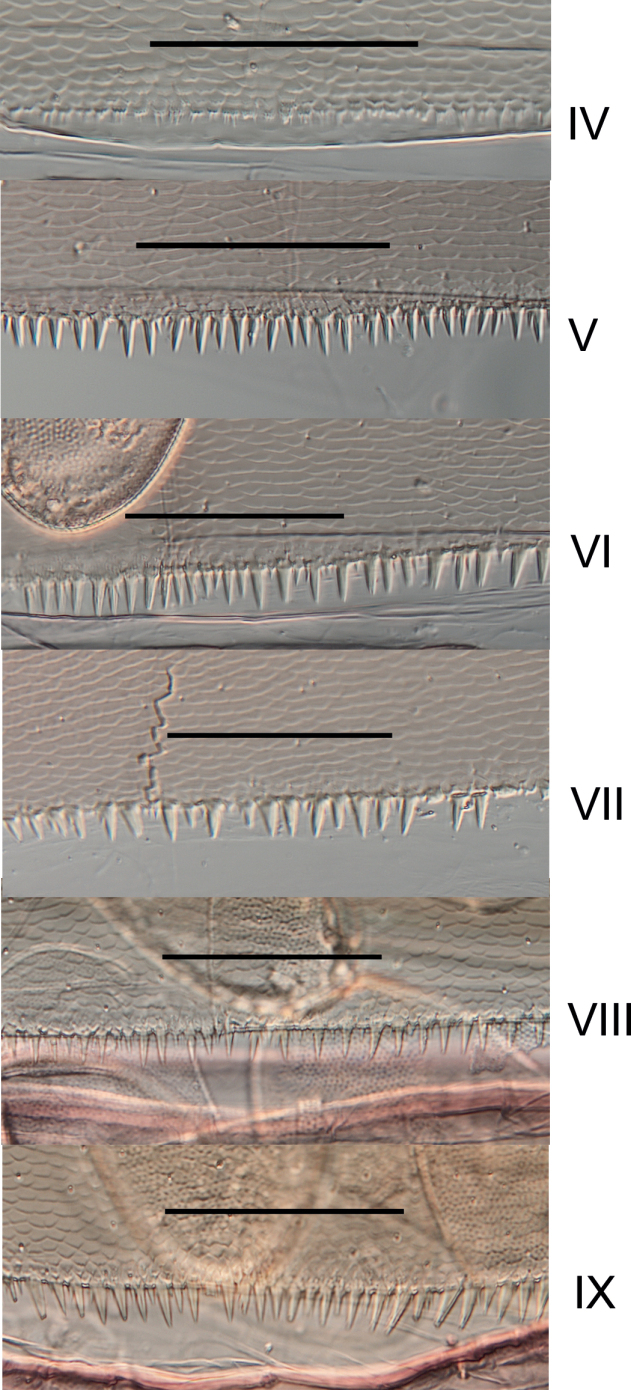
*Habrophlebiadakkii* sp. nov., posterior margin of abdominal tergites IV to IX of the nymph. Scale bar: 100 µm.

**Figure 7.**
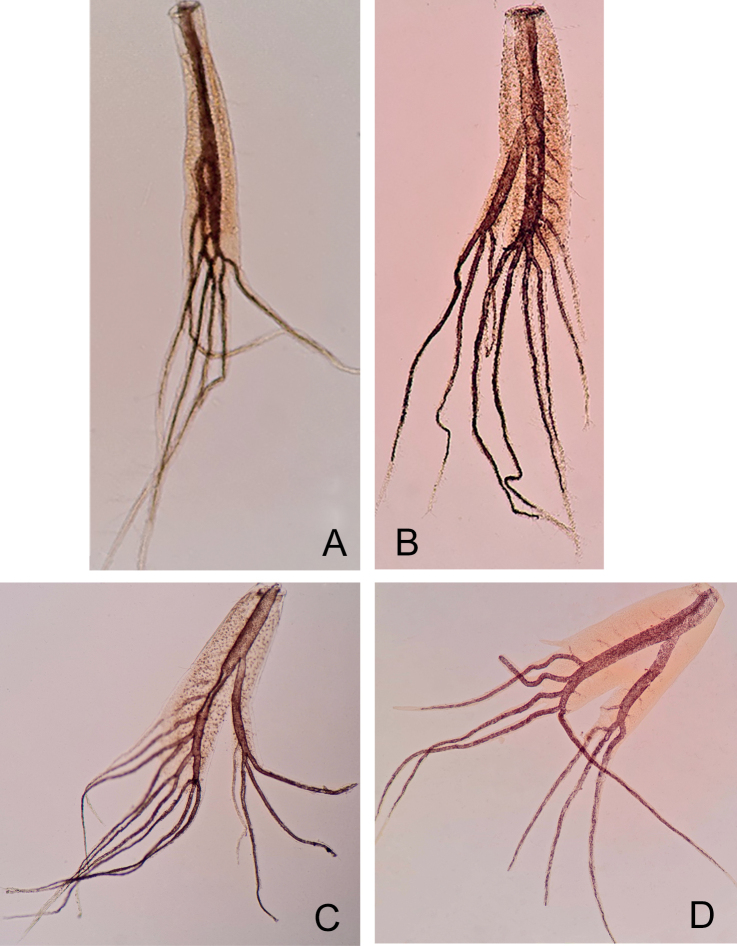
*Habrophlebiadakkii* sp. nov., nymphal gills **A** gill I **B** gill III **C** gill IV **D** gill VII.

**Figure 8.**
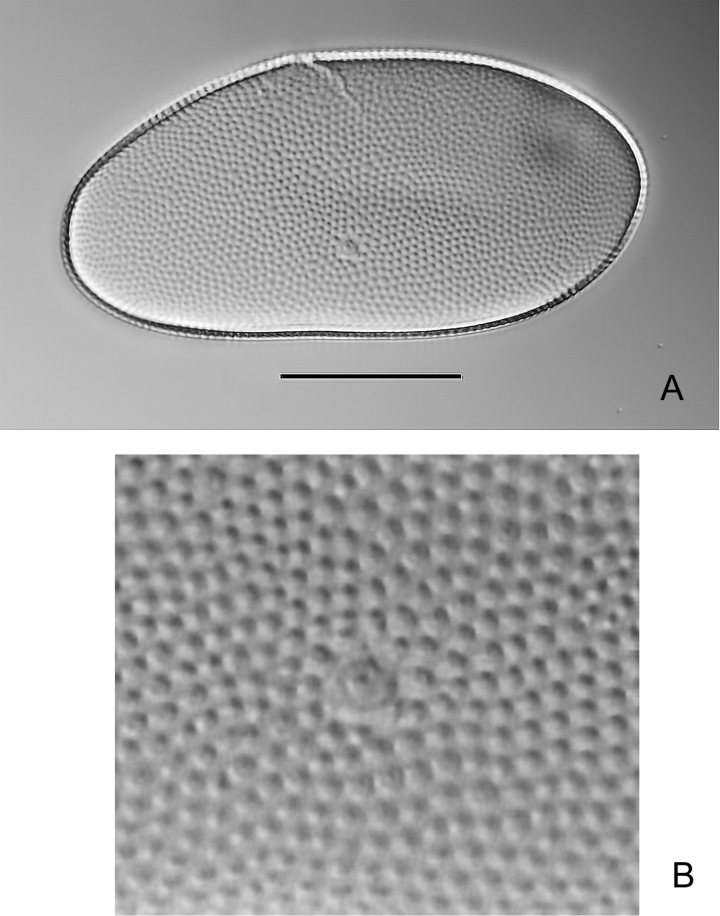
*Habrophlebiadakkii* sp. nov., egg **A** overview of the chorionic structure **B** detail of the chorion with micropyle. Scale bar: 50 µm.

Figs 2 , 3 , 4 , 5 , 6 , 7 , 8

Habrophlebia sp. in El Alami 2002; Khadri et al. 2017; El Bazi et al. 2017.
Habrophlebia sp.1 in El Alami et al. 2022a.

##### Material examined.

***Holotype***: one nymph in ethanol (GBIFCH01133087), Morocco, Chefchaouen Province, S4 Oued Amazithen, Loc. El Ouesteyine; 35°17.299'N, 4°55.267'W; alt. 483 m; 2.IX.2021; S. El Yaagoubi leg.; MZL. ***Paratypes***. Morocco, Chefchaouen Province, same data as holotype; 9 nymphs in ethanol (GBIFCH01133086); 1 nymph on slide (GBIFCH01144258-DNA); MZL; same locality as holotype; 19.IX.2014; Khadri leg.; 2 nymphs in ethanol (GBIFCH01133083); MZL • Chefchaouen Province, S7 Oued Harakat, Loc. Mezine village; 35°6.133'N, 5°20.767'W; alt. 740 m; 31.III.2021; S. El Yaagoubi leg.; 6 nymphs in ethanol (GBIFCH01133085); 1 nymph on slide (GBIFCH01144257-DNA); same locality, 29.II.2020; 1 nymph on slide (GBIFCH00970950); MZL • Chefchaouen Province, S5 Oued Sidi Yahia Aarab, Loc. Sidi Yahia Aarab; 35°17.179'N, 4°53.625'W; alt. 347 m; 18.VI.2014; M. El Alami leg.; 2 nymphs in ethanol (GBIFCH01133079); MZL; 4 nymphs in ethanol; 1 nymph on slide; LESCB; same locality, 27.XI.2021; S. El Yaagoubi leg.; 1 nymph in ethanol (GBIFCH01133080); 1 nymph on slide (GBIFCH01144259-DNA); MZL • Chefchaouen Province, S6 Oued Jnane Nich, Loc. Jnane Nich; 35°16.1856'N, 4°52.128'W; alt. 215 m; 12.VIII.2021; S. El Yaagoubi leg.; 3 nymphs in ethanol; LESCB • Chefchaouen Province, S8 Oued Ima sunna, Loc. Tanaqoub; 35°5.533'N, 5°23.604'W; alt. 684 m; 31.III.2021; S. El Yaagoubi leg.; 2 nymphs in ethanol (GBIFCH01133084); 1 nymph on slide (GBIFCH00970948-DNA); MZL; 1 nymph in ethanol; 1 nymph on slide; LESCB • Chefchaouen Province; S1 Oued Kelâa, Loc. Akchour; 35°14.333'N, 05°10.144'W; alt. 460 m; 17.IV.2008; M. El Alami leg.; 3 nymphs in ethanol (GBIFCH01133088); MZL; 13 nymphs in ethanol, 1 nymph on slide; LESCB • Chefchaouen Province, S2 Oued Talambote, Loc. usine électrique; 35°16.665'N, 5°13.46.171'W; alt. 129 m; 2.VI.2021; S. El Yaagoubi leg.; 20 nymphs in ethanol; LESCB • Chefchaouen Province, S3 Oued Laou, Loc. Afertane; 35°20.924'N, 5°11.241'W; alt. 55 m; 4.VI.2022; S. El Yaagoubi leg.; 20 nymphs in ethanol; LESCB.

• Tetouan Province, S9 Oued El Hamma, Loc. Jbel Laalam; 35°23.387'N, 5°29.953'W; alt. 200 m; 20.IV.2021; S. El Yaagoubi leg.; 6 nymphs in ethanol (GBIFCH01133076);1 nymph on slide (GBIFCH00970944-DNA); MZL; same locality; 20.II.2022; S. El Yaagoubi leg.; 9 nymphs in ethanol; 1 nymph on slide; LESCB • Tetouan Province, S10 Oued Tisgris, Loc. Hammadesh; 35°22.033'N, 5°32.033'W; alt. 505 m; 20.IV.2021; S. El Yaagoubi leg.; 8 nymphs in ethanol (GBIFCH01133077); 1 nymph on slide (GBIFCH00970946-DNA); MZL; same data; 7 nymphs in ethanol; LESCB • Tetouan Province, S11 Oued Taida, Loc. Beni idder; 35°22.102'N, 5°32.283'W; alt. 507 m; 16.VII.2021; S. El Yaagoubi leg.; 7 nymphs in ethanol (GBIFCH01133081); 1 nymph on slide (GBIFCH01144262-DNA); MZL; same locality; 15.V.2017; M. El Alami leg.; 4 nymphs in ethanol; LESCB.

• Larache Province, S14 Oued Stah, Loc. Tzroute; 35°16.583'N, 5°31.883'W; alt. 766 m; 2.V.2021; S. El Yaagoubi leg.; 16 nymphs in ethanol (GBIFCH01133078); 2 nymphs on slide (GBIFCH00970945-DNA, GBIFCH01144261-DNA); MZL; same data; 8 nymphs in ethanol; 2 nymphs on slide; LESCB; same locality; 18.VI.2022; S. El Yaagoubi leg.; 5 nymphs in ethanol; 1 nymph on slide; LESCB.

• Ouezzane Province, S13 Oued Qoub, Loc. Souk El Had; 35°1.283'N, 5°25.300'W; alt. 143 m; 11.IV.2021; S. El Yaagoubi leg.; 5 nymphs in ethanol (GBIFCH01133082); 1 nymph on slide (GBIFCH00970947-DNA); MZL; same data; 4 nymphs in ethanol; LESCB; same locality; 3.VI.2022; S. El Yaagoubi leg.; 2 nymphs in ethanol; LESCB • Ouezzane Province, S12 Oued Loukkos, Loc. Souk El Had; 35°1.350'N, 5°25.233'W; alt. 140 m; 11.IV.2021; S. El Yaagoubi leg.; 2 nymphs in ethanol; LESCB.

##### Description.

**Nymph. *Coloration and dimensions*.** Body length of final instar, excluding caudal filaments, 5.2 to 6.5 mm for male and 5.5 to 8 mm for female. Cerci as long as body length. General dark brown coloration with light brown to yellowish markings mainly on abdominal terga. The whole cuticle is shagreened.

***Head***. General coloration light brown; paler area between compound eyes and lateral ocelli; between ocelli, a dark brown mark not reaching the clypeus distally, and extending laterally in front of the compound eyes; vertex sutures yellowish, well visible (Fig. 2). Upper portion of male eyes reddish-brown. Antenna with pedicel greyish brown, scape and filament yellowish.

***Labrum*** rectangular (Fig. 3A, B), ca 2× wider than long; dorsal surface covered distally with scattered stout setae, proximally with long and thin setae; anterior margin with a row of stout, long and spatulate setae medially; emargination narrow, U-shaped with six flat denticles (Fig. 3C); ventral surface with two bunches of stout setae medially.

***Mandibles*** similar to other *Habrophlebia* species (Fig. 3D, E).

***Maxilla*** (Fig. 4) stocky, subapical row of 6 or 7 pectinate setae (Fig. 4A); maxillary palp with three segments, segment 1 and 2 subequal in length, and longer than segment 3; segment 3 triangular a little bit 1.5× longer than wide at base; all setae on palp segments stout and entire (Fig. 4B).

***Hypopharynx*** with highly developed superlinguae terminated by a membranous digitation (Fig. 4D).

***Labium*** (Fig. 4C) with rhomboid glossae, outer margin and apex covered by short, broad setae; paraglossae enlarged laterally, covered with fine and long setae on the dorsal surface; with long, thick setae on the outer margin; labial palp with three segments, inner margin of segment 1 highly dilated near the middle, about 0.8× longer than the maximum width, segments 2 as long as segment 3, ca 0.7× length of segment 1; segment 3 ca 1.6× longer than wide at base, conical shape and the inner margin with one row of longer and robust setae.

***Thorax*.** Pro- and mesonotum yellowish to light brown, with greyish brown maculae, on medium and lateral margins (Fig. 2A).

***Legs*** light to medium brown; dorsal surface of femora almost entirely washed with greyish brown macula; tarsi and tibiae generally lighter, except sometimes in mature nymphs.

***Fore legs*** (Fig. 5A) femora elongated, ca 1.2× longer than wide, upper surface covered with long, entire and pointed setae; fore tibiae subequal in length to femora, outer margin with thin and long setae, inner margin with several rows of long stout and feathered setae especially near apex; tarsi 0.4× length of tibiae, outer margin with long and thin setae, inner margin with long and pointed feathered setae.

***Middle legs*** similar to fore legs, femora ca 2.5× longer than wide, dorsal surface of femora with more numerous and slightly longer stout and pointed setae; tibiae and femora of subequal length; tarsi 0.5× length of tibiae.

***Hind legs*** (Fig. 5B) with femora 2.8× longer than wide, dorsal surface covered with stout, long, pointed and feathered setae (Fig. 5C); ventral surface with few feathered setae; hind tibiae as long as hind femora, outer margin with scattered stout, pointed setae; inner margin with stout, pointed, scattered setae; tarsi 0.4× length of tibiae, outer margin with long and thin setae, inner margin with long, stout, pointed setae. Claws (Fig. 5D) of all legs slightly hooked, with 15 to 18 long, thin, pointed denticles that decrease in size from the apex to the tarsus.

***Abdomen*.** Grey to dark brownish terga with characteristic light markings (Fig. 2A). Tergites I-II with a dark brown color, terga III–IX with two sublateral elongated dark brown maculae, joining on the posterior margin, leaving two lateral light areas pronounced on tergites III–VIII; posterior marking sometimes absent on tergites IX−X. Sternite I entirely greyish brown (Fig. 2B); sternites II−VIII yellowish with lateral, anterior and posterior bands brown, and two parasagittal nervous ganglia greyish browns; sternite IX lighter brown, in male nymphs with genitalia well visible and styliger plate dark brown. Posterolateral expansions only on segments VIII and IX. Posterior margin of tergite IX with well-developed narrow and pointed spines, ca 2–3× longer than wide at base (Fig. 6); shape and size of posterior spines on abdominal segments V to VIII similar, a little bit smaller than those on segment IX; tergites I–IV with barely visible spines (Fig. 6).

***Gills*** present on segments I–VII; all gills long and large; first gill (Fig. 7A) with dorsal lamella bearing 3 or 4 filaments, ventral lamella with 2–3 filaments, gills II−VII (Fig. 7B–D) with 3–4 and 6–9 filaments on the ventral and dorsal lamella respectively. Cerci and paracercus yellowish brown, medium brown in mature nymphs (Fig. 2A–C).

**Eggs** ovoid, ca 155 µm x 80 µm, without ribs; chorion surface regularly decorated by numerous and small granulations (Fig. 8).

**Imago.** Unknown.

##### Etymology.

The first author dedicates this species to her former mentor, Professor Dakki Mohamed. He contributed significantly to her training and specialization in the hydrobiological study of Moroccan streams.

##### Molecular analysis.

**Figure 9.**
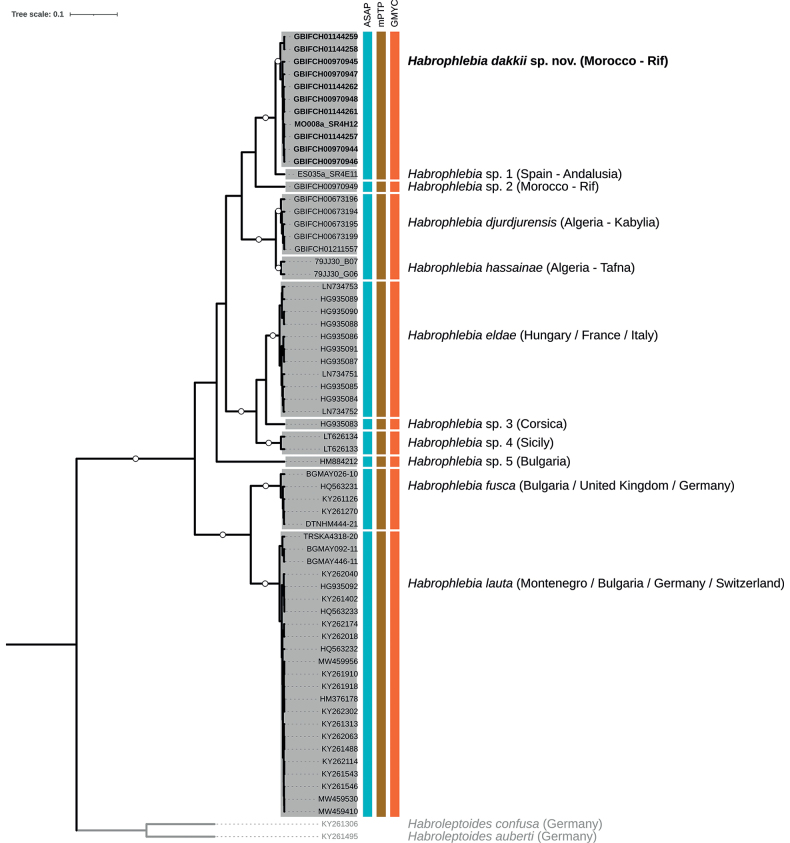
Bayesian (BEAST) maximum clade credibility COI tree of the genus *Habrophlebia* in the West Palearctic. Branch ends labelled with GBIF or 79JJ30 codes indicate newly sequenced specimens; the other codes correspond to sequences obtained from various sources (see material and methods). Colored vertical boxes indicate species delimitation hypothesis (MOTUs) according to the ASAP, mPTP, and GMYC methods. For each MOTU, the corresponding species names (where available) and the country (-region) of origin is provided, with the newly described species and associated GBIF codes specified in bold. Circles on branches indicate Bayesian posterior probabilities > 0.9. The outgroup branches are presented in grey, along with their corresponding labels and species names.

The COI data set was > 97% complete and included 26% of parsimony informative sites. Pairwise COI distances across all sequences ranged from 0% to 18.2%. All species delimitation methods were fully congruent in delimiting nine *Habrophlebia* MOTUs (Fig. 9). Among these, the 11 sequences from the new species formed a strongly supported monophyletic clade, closely related to the sequence of *H.* sp. 1 from Spain (Andalusia) from which it exhibits a minimal p-distance of 0.9%. According to our species delimitation methods, both lineages were grouped into a single MOTU. Similarly, the sequences of *H.djurdjurensis* and *H.hassainae* formed two distinct, well-supported, sister clades, that were merged into the same MOTU (Fig. 9). The overall mean p-distance within MOTUs was 1.0% (mean range: 0.4%–1.9%), while the overall mean p-distance between MOTUs was 12.9% (mean range: 7.0%–16.9%). The maximum p-distance within MOTUs varied from 1.3% (*H.* sp. 4) to 4.2% (*H.djurdjurensis* + *H.hassainae*). The minimum p-distance between MOTUs ranged from 6.7% (*H.* sp. 3–*H.eldae*) to 12.3% (*H.fusca*–*H.lauta*). The maximum p-distance within the new species was 1.5%, whereas it was 1.8% for both *H.djurdjurensis* and *H.hassainae*. The minimum p-distance between the new species and *H.* sp. 1 was 0.9%, and between *H.djurdjurensis* and *H.hassainae* was 2.1%.

## ﻿Discussion

Until the late 1970s, only two species of Ephemeroptera were known from the Rif, due to the works of Eaton (1899), Navás (1922, 1929, 1935), Lestage (1925) and Kimmins (1938). This fauna corresponded to two Baetidae recorded in the regions of Tétouan and Chefchaouen [*Baetisrhodani* (Pictet, 1843) and Procloeoncf.concinnum]. Since then, a large number of surveys have been carried out to augment this initial list with an additional 35 species, resulting in a total species richness of 37 taxa, which constitutes more than 68% of Morocco’s Ephemeroptera (Dakki and El Agbani 1983; Giudicelli and Dakki 1984; El Alami and Dakki 1998; El Alami et al. 2000, 2022a; Kaltenbach et al. 2022; Gattolliat et al. 2023; El Alami et al. 2023; El Yaagoubi et al. 2023). These investigations have collected no less than three new species from the Rif, including *Habrophlebiadakkii* sp. nov.

### ﻿Morphology characteristics

The main characters used to distinguish the hitherto known species from the new one, are presented in Table 2. At the egg stage, *Habrophlebiadakkii* sp. nov. presents a unique chorionic arrangement among *Habrophlebia* species, composed of fine granulations evenly distributed on the whole surface, without any other attachment structures (ribs, polar caps). *Habrophlebiadakkii* sp. nov. is most similar to *H.eldae*, especially concerning the abdominal spines on the posterior margin of abdomen, although those of *H.eldae* are larger than those of *H.dakkii*. The latter mainly differs from the former by the number of gill filaments. *Habrophlebiadakkii* can be easily separated from the other Maghrebian species; from *H.hassainae* by the number of denticles on the claw, by the number of gill filaments, and by the shape of posterior abdominal spines on tergites V; from *H.djurdjurensis* also by the shape of posterior abdominal spines on tergites V-VIII; and from *H.vaillantorum* by the number and the size of denticles on the tarsal claw, the number of gill filaments, and by the shape of posterior abdominal spines on tergites V-IX.

**Table 2.** Taxonomic criteria differentiating nymphs and eggs of Western Palaearctic *Habrophlebia* species: (0) Present study; (1) Thomas et al. 1999; (2) Wagner at al., 2007; (3) Mazzini and Gaino 1985 (H.eldae sub. nom. H.fusca); (4) Zrelli et al. 2011; (5) Alba-Tercedor 2000; (6) Benhadji et al. 2018; (7) Kechemir et al. 2020; (8) Biancheri 1959; (9) Kimmins 1954; (10) Landa 1969; (11) M. Sartori, pers. obs.; (12) Jacob and Sartori 1984; (13) Bauernfeind and Soldán 2012; (14) Thomas and Bouzidi 1986; (15) Righetti 2020; (16) Belfiore and Gaino 1985.

Character	*H.fusca*	*H.lauta*	*H.eldae*	*H.consiglioi*	*H.antoninoi* (5)	*H.vaillantorum*	*H.hassainae* (6)	*H.djurdjurensis* (7)	*H.dakkii* sp.nov. (0)
Egg chorionic structure	ribs long, punctuated (1)	ribs long, not punctuated (2)	ribs long, slightly punctuated (3)	ribs barbed, long , punctuated (3,4)	ribs forming a reticulated mesh	ribs long, not punctuated (1)	ribs short, not punctuated	ribs long, not punctuated	without ribs, entirely covered with small granulations
Position of the costal process of hind wing	middle (9)	middle (10)	middle (11)	middle (8)	distal	middle (1)	middle	middle	middle
Bristles on upper face of hindfemur	truncated, entire (12)	pointed, entire (12)	pointed, fringed (12)	pointed, fringed (12)	?	pointed, entire (13)	pointed, fringed	pointed, fringed	pointed, fringed
Number of denticles on claws	11–13 (11)	14–16 (11)	14–17 (11, 15)	15–17 (11)	?	13–16 (1, 14)	18–22	15–18	15–18
Size of distal denticles on claws	normal (11)	normal (11)	normal (11)	normal (11)	?	reduced (1, 14)	normal	normal	normal
Number of filaments on dorsal (costal) and ventral (anal) lamellae of gills II-VI	7–8; 3–4 (13)	5–7; 4–5 (13)	3–7; 2–4(13)	3–6; 1–3 (16)	?	5–6; 4–5 (1, 14)	9–12; 5–8	8–11; 4–7	6–9; 3–4
Shape and size of posterior spines on abdominal segment IX	truncated, wider than long (1, 2, 16)	triangular, as long as wide at base (2, 16)	triangular, 2–3× longer than wide at base (2, 16)	triangular, 1.5–2× longer than wide at base (16)	?	minute, needle-shaped (1)	triangular, 2–3× longer than wide at base	lanceolate, 2–3× longer than wide at base	triangular, 2–3× longer than wide at base
Shape and size of posterior spines on abdominal segment VIII	truncated, wider than long (16)	triangular, as long as wide at base (16)	triangular, 2–3× longer than wide at base (16)	triangular, 1.5–2× longer than wide at base (16)	?	? (probably minute, needle-shaped)	triangular, 2–3× longer than wide at base	minute, needle-shaped	triangular, 2–3× longer than wide at base
Shape and size of posterior spines on abdominal segment VII	truncated, wider than long (16)	triangular, as long as wide at base (16)	triangular, 2–3× longer than wide at base (16)	triangular, 1.5–2× longer than wide at base (16)	?	minute, needle-shaped (1)	triangular, 2× longer than wide at base	minute, needle-shaped	triangular, 2–3× longer than wide at base
Shape and size of posterior spines on abdominal segment V	truncated, wider than long (16)	triangular, as long as wide at base (16)	triangular, 2–3× longer than wide at base (16)	triangular, as long as wide at base (16)	?	? (probably minute or absent)	minute, needle-shaped	minute, needle-shaped	triangular, 2–3× longer than wide at base

### ﻿Genetic characteristics

While the 11 sequences from the new species group together in a well-supported COI clade (Fig. 9), they are sister to the sequence of *H.* sp. 1 from Spain (Andalusia) obtained from a young nymph, showing a minimal p-distance of 0.9% between both lineages. Such a genetic distance is typically found within species, as reported in insects in general (e.g., Virgilio et al. 2010) and especially in mayflies (e.g., Ball et al. 2005; Morinière et al. 2017). This observation is further corroborated by the results obtained from the three species delimitation methods. To conclusively determine whether these lineages represent distinct species and potentially establish the new species as a Moroccan endemic or if they constitute a single species with an extended geographic range, additional investigations in the Iberian Peninsula are imperative with new material. A similar situation occurred with the recently described baetid species *Baetisrifensis* El Yaagoubi, Vuataz & Gattolliat, 2023 from Morocco. Its closest COI sequences were from three specimens sampled in the Iberian Peninsula, but in this case, the minimum p-distance between the Moroccan and Iberian lineages was higher (3.2%; El Yaagoubi et al. 2023).

Also interesting is the close genetic relationship between *H.hassainae* and *H.djurdjurensis*; both species are only separated by a minimum genetic distance of 2.1%, which suggests they could belong to the same species. Morphologically however, both species differ by a number of characters (Table 2), among which are the shape and size of spines on the posterior margin of abdominal tergites, the number of gill filaments and the number of denticles on the claw. Such a low COI divergence between species, possibly indicating recent reproductive isolation, has been sporadically documented in animals (Hebert et al. 2003), including mayflies (e.g., Morinière et al. 2017), and appears to be relatively frequent among closely related species pairs of stoneflies (Vuataz pers. obs.)

Our COI analyses have identified a new *Habrophlebia* lineage in the Rif region, labeled as *H.* sp. 2 (Fig. 9). Initial morphological examinations suggest it may be a new species, which will be further investigated in a future study. Additionally, we could not assign names to several other COI clades. While *H.* sp. 3 from Corsica and *H.* sp. 4 from Sicily were previously identified as *H.eldae* in Gattolliat et al. (2015) and Tenchini et al. (2018), respectively, the COI distances between those three lineages (see Fig. 9) are more indicative of distinct species (with minimum p-distance of *H.eldae*–*H.* sp. 3: 6.7%; *H.eldae*–*H.* sp. 4: 8.5%; *H.* sp. 3–*H.* sp. 4: 7.7%), as pointed out by Tenchini et al. (2018). Similarly, *H.* sp. 5 from Bulgaria, presently labeled as *H.fusca* in GenBank, clearly differs from *H.fusca* and all other lineages in our dataset. The p-distance to its nearest neighbors, *H.eldae* and *H.lauta* (12.2%), strongly supports *H.* sp. 5 as a distinct, new species. These results emphasize the need for more taxonomic research on this genus in Europe.

### ﻿Distribution and ecology

This species is widely distributed in the Rif, where it occupies a large range of biotope types, spanning from sea level up to an altitude of 780 m. It also tolerates wide variations in water conductivity (35 to 1112 µS/cm). *Habrophlebiadakkii* has a clear preference for poorly mineralized headwaters with moderate current velocity. The substrate is characterized by pebbles, gravel, sand and silt covered in some places with algae and submerged macrophytes, which provide excellent refuge for nymphs when the current is stronger.

The same remark was made by Gagneur and Thomas (1988) concerning *Habrophlebiahassainae* (sub. nom. H.gr.fusca) found in Algeria.

Since this species has been found in localities on the Oued Ouergha, which is the Rif tributary of the Oued Sebou, we believe that its presence in the Haut Sebou (Middle Atlas) and the coastal Meseta is highly probable.

Nymphs of *Habrophlebiadakkii* sp. nov. can be found all year long, but are most abundant in spring, when temperatures are optimal for their development.

## Supplementary Material

XML Treatment for Habrophlebia dakkii

## ﻿Additional information

### Author ORCIDs

Majida El Alami https://orcid.org/0000-0003-2664-646X

Laurent Vuataz https://orcid.org/0000-0001-9193-8683

Sara El Yaagoubi https://orcid.org/0000-0003-1860-6433

Michel Sartori https://orcid.org/0000-0003-3397-3397

### Data availability

All of the data that support the findings of this study are available in the main text.
